# Microstructure and Mechanical Properties of Cast Al-Si-Cu-Mg-Ni-Cr Alloys: Effects of Time and Temperature on Two-Stage Solution Treatment and Ageing

**DOI:** 10.3390/ma16072675

**Published:** 2023-03-28

**Authors:** Lairong Xiao, Huali Yu, Yiwei Qin, Guanqun Liu, Zhenwu Peng, Xiaoxuan Tu, Heng Su, Yuxiang Xiao, Qi Zhong, Sen Wang, Zhenyang Cai, Xiaojun Zhao

**Affiliations:** 1 School of Materials Science and Engineering, Central South University, Changsha 410083, China; 2 Key Laboratory of Non-Ferrous Metal Materials Science and Engineering, Ministry of Education, Central South University, Changsha 410083, China

**Keywords:** Al-Si alloys, microhardness, solution treatment, ageing treatment, mechanical properties

## Abstract

Ameliorating the high-temperature performance of cast Al-Si alloys used as engine components is essential. The effects of different T6 heat-treatment processes on the microstructure and mechanical properties of cast Al-Si-Cu-Mg-Ni-Cr alloys were investigated in the present study. The results demonstrate that, under the optimal solution treatment conditions of 500 °C for 2 h and 540 °C for 4 h, the T-Al_9_FeNi phase was present in the alloy, and the roundness of primary Si and the aspect ratio of eutectic Si in the alloy reached valley values of 1.46 and 2.56, respectively. With increasing ageing time at 180 °C, the tensile strength significantly improved, while the microhardness first increased and then decreased. When the ageing time was 4 h, microhardness reached a peak value of 155.82 HV. The fracture characteristics changed from quasi-cleavage to the coexistence of quasi-cleavage and dimples. After heat treatment, the high-temperature tensile properties of the alloy improved, which is a significant advantage compared to the as-cast alloy. The stable Al_3_Ni and Al_9_FeNi phases inhibited the cracking of the alloy at 350 °C.

## 1. Introduction

Al-Si alloys have become attractive lightweight materials owing to their low density, high specific strength, good casting properties, and corrosion resistance. Cast heat-resistant Al-Si-Cu-Mg-Ni alloy has been widely used in the armament, marine, aviation, aerospace, and automotive industries, especially in pistons, brake discs, and even cylinder heads for automotive engines [1,2,3]. However, the complex operating environment of engines requires that the materials used for pistons retain good mechanical properties in the temperature range of 200–400 °C. The mechanical properties of Al-Si alloys tend to deteriorate abruptly at temperatures above 250 °C owing to the rapid coarsening of the second phase at high temperatures. Moreover, the needle-like Al_5_FeSi brittle phase formed in the alloy tends to cause stress concentration and reduce the mechanical properties of the Al-Si alloy. Consequently, this remains a challenge for Al-Si alloys [4,5,6,7,8].

Considerable attention has been paid to the exploration of more thermally stable phases of cast Al-Si-Cu-Mg-Ni alloys to improve their mechanical properties [9,10,11,12,13,14,15]. The addition of alloying elements is an effective method owing to the formation of resistant phases, such as ε-Al_3_Ni, M-Mg_2_Si, δ-Al_3_CuNi, γ-Al_7_Cu4Ni, θ-Al_2_Cu, Q-Al_5_Cu_2_Mg_8_Si_6_, T-Al_9_FeNi, and π-Al_8_Si_6_FeMg_3_ [16,17]. Sha et al. [18] reported that Cr-, Zr-, Ti-, and V-containing dispersoid phases improved the strength of alloys at high temperatures. Feng et al. [19] studied the ultimate tensile strength and yield strength of an alloy with an Al_3_Ni phase at higher temperatures. Li et al. [20] reported that the tensile strength of Al-Si alloys at 250 °C increased from 157 to 199 MPa owing to the presence of the 3.2–6.4% Q-Al_5_Cu_2_Mg_8_Si_6_ phase. The addition of Cr could transform the needle-like β-Al_5_FeSi phase to the fishbone α-Al (Fe, Cr) Si phase, which could neutralise the negative effect of Fe as an impurity. Further, Yang et al. [21] observed that the addition of Cr can also increase the elongation of the alloy. Zhan et al. [22] reported that the microhardness of an Al-Si-Mg alloy after quenching can be increased by more than 10 HV by adding Cr. Li et al. [23] showed that the HT tensile strength of an Al-13Si-4Cu-1Mg-3Ni alloy with 0.8% Fe and 0.5% Cr increased from 78 to 98 MPa at 350 °C.

In addition, heat treatment influences the size, morphology, and distribution of the second phases and the precipitation strengthening, thus leading to an improvement in the mechanical properties of the alloy. Khisheh et al. [24] reported that T6 treatment significantly improves the fatigue life of unaged alloys under thermo-mechanical fatigue (TMF) loading. Luna et al. [25] proved that a two-stage solution cycle treatment is more effective than a conventional single-stage solution treatment in improving the mechanical properties of the alloy. Sun et al. [26] reported that the solid solution temperature of the Al-15% Mg_2_Si (−1% Cu) alloy significantly affected the formation of precipitated phases and age-hardening behaviour. Although the evolution of a second phase in the heat treatment of Al-Si-Cu-Mg-Ni alloys has been extensively studied, studies on the influence of heat treatment parameters on the morphology distribution of the second phase in cast alloys with transition elements are limited. In addition, conventional single-step solution treatment at a temperature lower than the melting point of the Cu-rich phase results in insufficient solution strengthening of the alloy. 

Therefore, Al-Si-Cu-Mg-Ni alloys containing 0.2% trace Cr were prepared in this study. The effects of the time and temperature of the two-stage solution treatment and ageing on the microstructure and mechanical properties of the alloys were investigated. The role of intermetallic phases in the improved strength of alloys at room and higher temperatures was analysed and the appropriate conditions for the heat treatment of alloys were determined. The research in this paper focused on reducing the weight of heavy truck engines. Making Al-Si-Cu-Mg-Ni-Cr alloys for pistons played a better role in practical applications.

## 2. Experimental Procedures

### Preparation of Materials

**Figure 1:**
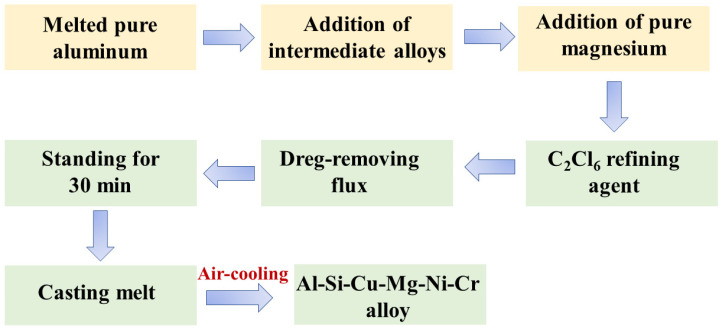
Melting process flow chart.

The alloys were prepared using 99.8% commercially pure Al, 99.9% pure Mg, Al-20% Si, Al-50% Cu, Al-10% Ni, Al-10% Cr, Al-3% Ti, and Al-10% Zr intermediate alloys (wt.%). All metal materials were purchased from the Hunan Rare Earth Metal Materials Research Institute, Hunan, China. First, pure Al ingots were placed in a graphite crucible in an electric resistance furnace at 750 °C. The master alloys were then added to the melt. The melt refining process was degassed using C_2_Cl_6_ and dreg-removing flux. Finally, the cast alloys were obtained after pouring the molten alloy into a preheated metal die and air-cooling it to room temperature. The specific experimental process was shown in Figure 1. Table 1 lists the chemical compositions of the cast alloys measured using inductively coupled plasma (ICP) atomic emission spectrometry.

**Figure 2:**
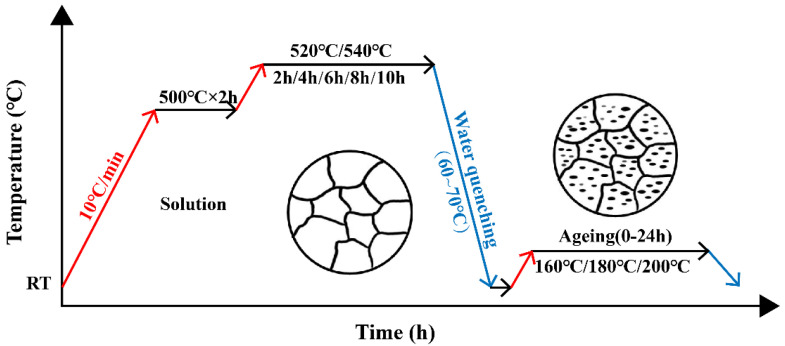
Schematic diagram of the heat treatment process.

After casting, the Al-Si-Cu-Mg-Ni-Cr alloy was subjected to a T6 heat treatment. Solution heat treatment (SHT) was conducted in two steps in a chamber resistance furnace with a temperature deviation of less than 5 °C. Following water quenching, the experimental alloys were immediately aged at different temperatures for various periods. The specific heat-treatment process is shown in Figure 2. 

The phase transition temperatures of the as-cast and solution-treated samples were analysed using differential scanning calorimetry (DSC, DSC204F1, NETZSCH, Selb, Germany) in the range of 30–600 °C under high-purity argon gas at a heating rate of 10 K/min. The phase compositions of the alloys were identified using X-ray diffraction (XRD; D/Max 2500, Rigaku, Tokyo, Japan) with Cu K_α_ radiation at 30 kV and 100 mA. The diffraction angle at which the X-rays hit the sample varied from 10° to 80° at a scanning rate of 8°/min. The microstructure and fracture morphologies were characterised using metallographic microscopy (DMIL MLED, Leica, Wetzlar, Germany) and scanning electron microscopy (SEM, FEI Sirion 200, FEI, Hillsboro, IL, USA). The elemental concentration profile of the microstructure was determined using energy-dispersive X-ray spectroscopy (EDS). The elemental distribution was analysed using electron probe microanalysis (EPMA, JEOL JXA-8230, JEOL Ltd., Tokyo, Japan). Image-Pro Plus 6.0 was used to analyse the roundness (*R*_a_) of primary Si and the aspect ratio of eutectic Si of the alloy. The *R*_a_ values of the primary Si particles are calculated using Equation (1):

(1) $$R_{a}=\frac{p^{2}}{4\pi A}$$

where *p* is the perimeter and *A* the area of each eutectic Si particle. 

The Vickers microhardness of the polished sample surface was tested according to ASTM C1327-15, and determined using a computerised microhardness tester (Shimadzu HMV-2T, Tokyo, Japan) at a load of 1 kg for a dwell time of 15 s. Seven indentations were made on each sample, and the average microhardness values were reported. 

**Figure 3:**
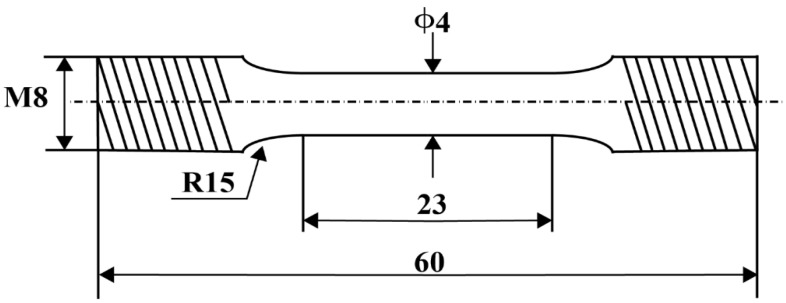
Schematic diagram and dimensions of the tensile samples (dimensions are in millimetres).

The geometry and dimensions of the sample for the tensile tests according to GB/T 228.1-2010 and GB/T 2288.2-2015 are shown in Figure 3. Room-temperature and high-temperature tensile tests were carried out at 0.1 mm/min using a tensile testing machine (DNS100, Cmc test equipment company, Beijing, China). The high-temperature tensile tests were performed at 350 °C. After the temperature was increased to the test temperature, the test was carried out after a 30 min holding period. To ensure the reliability of the measured data, three specimens were tested in each group and the average values were reported. 

## 3. Results and Discussion

### 3.1. Optimization of the Solid Solution Process

**Figure 4:**
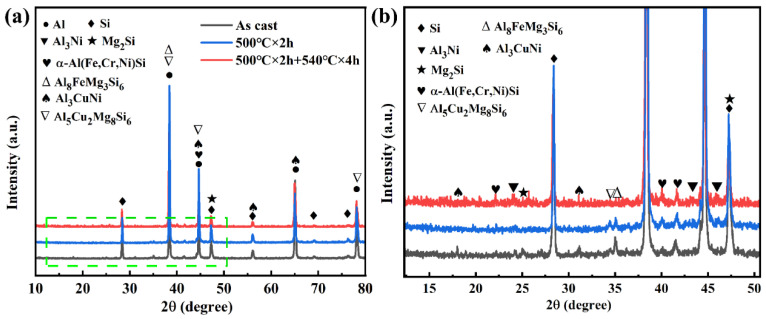
XRD patterns of the alloys: (**a**) at different states, (**b**) enlargement of the boxed area in (**a**).

Figure 4 shows the XRD patterns of eutectic Al-Si alloys under as-cast and different SHT conditions. The main phases were α-Al, Si, δ-Al_3_CuNi, Q-Al_5_Cu_2_Mg_8_Si_6_, α-Al (Fe, Cr, Ni) Si, Mg_2_Si, ε-Al_3_Ni, and π-Al_8_FeMg_3_Si_6_. In Figure 4a, the diffraction peak intensities of the δ-Al_3_CuNi and Q-Al_5_Cu_2_Mg_8_Si_6_ phases decreased after the two-stage SHT of the alloys compared to the as-cast and single-stage SHT conditions. When Cu was dissolved in the matrix, the Cu-rich phase on the surface of the matrix decreased, and the Mg_2_Si phase was partially dissolved. The magnified view of the XRD patterns in Figure 4b shows that the variation in diffraction peak intensities of the α-Al (Fe, Cr, Ni) Si and Al_3_Ni phases is insignificant with changing SHT conditions. 

**Figure 5:**
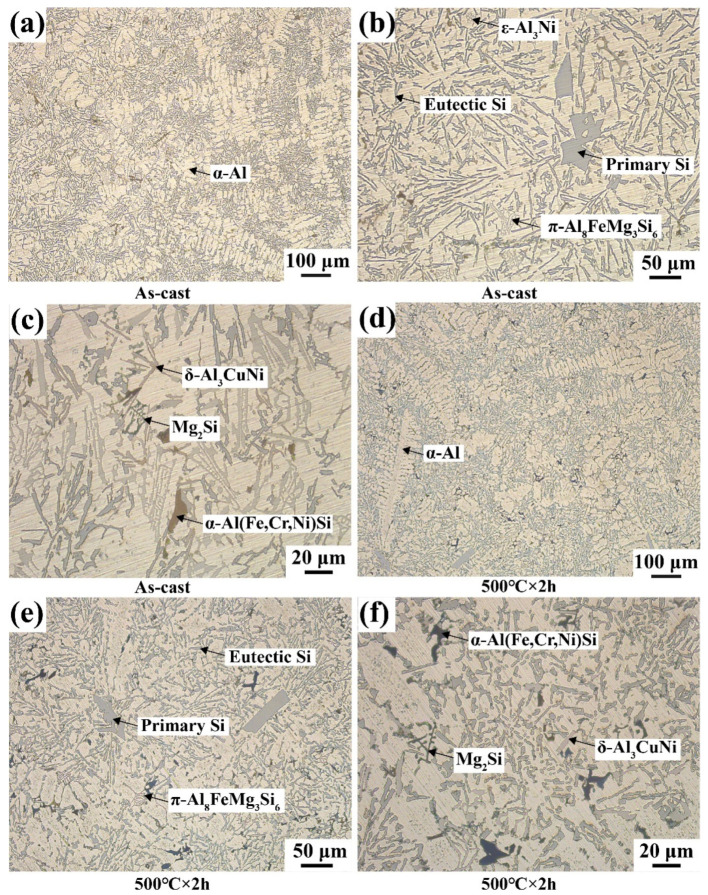
Optical micrographs of the alloys at different states: (**a**–**c**) as cast, (**d**–**f**) 500 ℃ for 2 h.

Figure 5 shows optical micrographs of the alloy in the as-cast and single-stage SHT conditions at 500 °C for 2 h. The microstructure included α-Al (Figure 5a,b) and different secondary phases characterised by reticulated Al_3_Ni (Figure 5c), fishbone α-Al (Fe, Cr, Ni) Si, black skeletal Mg_2_Si, hanky π-Al_8_FeMg_3_Si_6,_ and needle-like δ-Al_3_CuNi (Figure 5d,f). The mechanical properties of cast alloys are closely related to microstructural parameters, including the grain size, morphology of eutectic Si, and shape and distribution of intermetallic compounds. Primary Si typically exhibits an irregular blocky structure. After single-stage SHT, R_a_ of the primary Si in the as-cast state decreases from 4.3 to 3.88 with edge passivation. Few needle-like eutectic Si particles begin to fragment, and the aspect ratio decreases from 3.73 to 3.56. Furthermore, the intermetallic compounds showed no significant change after the single-stage SHT. 

**Figure 6:**
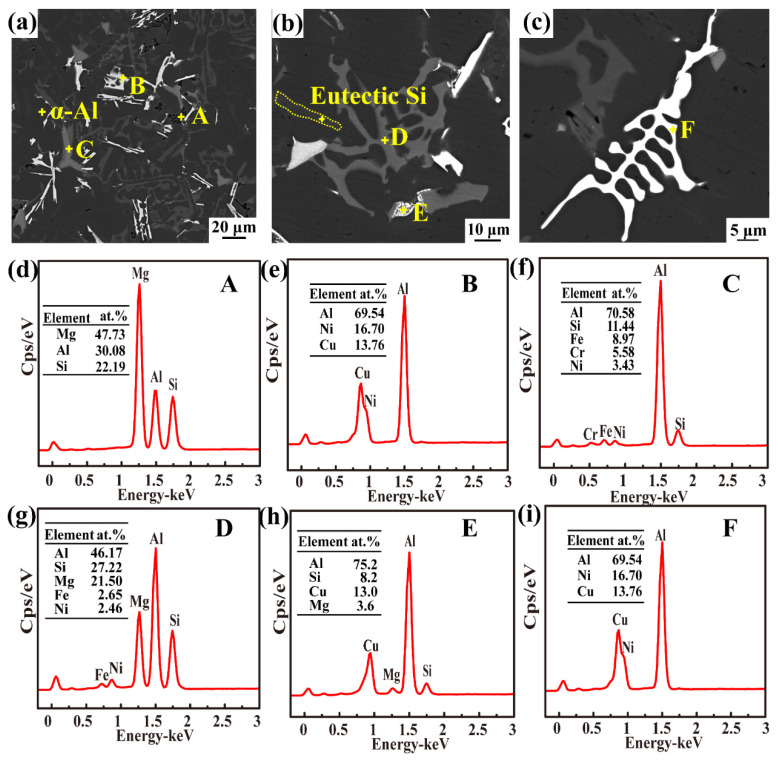
SEM morphology and EDS analysis of as-cast Al-Si alloy: (**a**–**c**) SEM morphologies, (**d**–**i**) EDS analysis result of Point A–F in (**a**–**c**).

Figure 6 shows the main intermetallic compounds in the as-cast alloy. As shown in Figure 6a, the white needle-like structure of the Al_3_CuNi phase (Figure 6e) and the dark grey Chinese-script π-Al_8_FeMg_3_Si_6_ phase (Figure 6g) are distributed in the matrix. Meanwhile, Cr mainly formed light grey α-Al (Fe, Cr, Ni) Si (Figure 6f). A greyish-white bulk Q-Al_5_Cu_2_Mg_8_Si_6_ phase (Figure 6h) was detected by XRD in combination with EDS analysis, as shown in Figure 6b). Above 0.6 wt.% Mg content is mainly used to form the π-Al_8_FeMg_3_Si_6_ phase, and the excess Mg forms Mg_2_Si (Figure 6d). When the Fe/Ni ratio was less than one, the formation of the Al-Cu-Ni ternary phase was promoted [27]. A bright white skeletal ε-Al_3_Ni phase (Figure 6i) is observed in Figure 6c. As a transition element, Ni has a low solubility in an α-Al solid solution. Ni-rich precipitation particles have a high melting point and superior stability, making them an ideal core for refining the grain size of as-cast alloys and improving their mechanical properties. 

**Figure 7:**
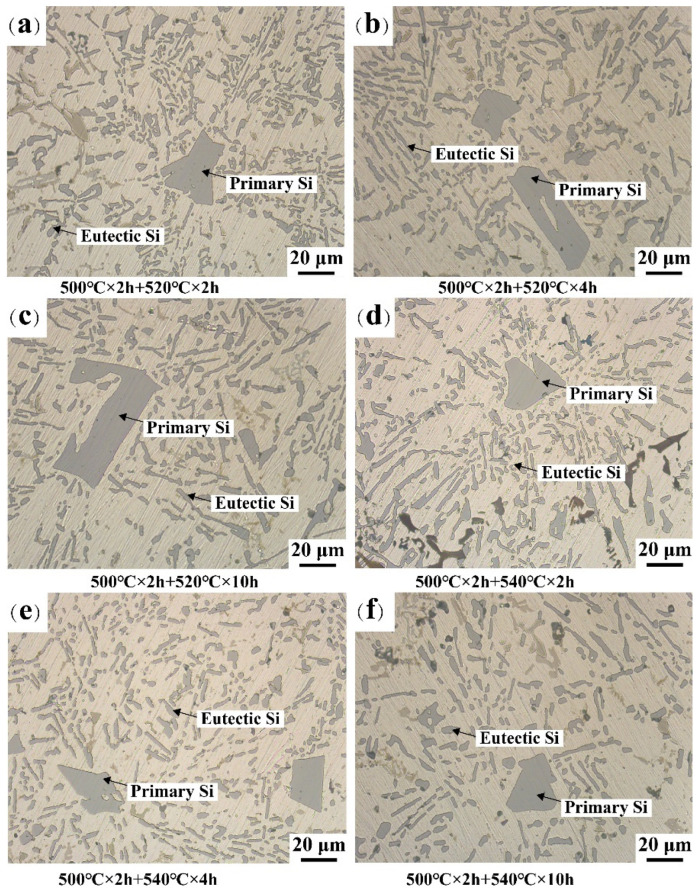
The optical micrographs of Al-Si alloys with different SHT: (**a**–**c**) 500 °C for 2 h and 520 °C, (**d**–**f**) 500 °C for 2 h and 540 °C.

Figure 7 presents the morphologies of eutectic Si and primary Si at different SHT conditions. With the increase in solution time, eutectic Si particles dissolve from the long needles and split into relatively small particles (Figure 7a,b) at the second solution temperature of 520 °C. After the second solid solution treatment at 520 °C for 10 h (Figure 7c), the eutectic Si particles started to become spherical. Simultaneously, the morphology of the primary silicon particles was edge passivated. After the solid solution process at 540 °C for 2–4 h, the morphology of the eutectic Si particles changed from strip to short rod and spherical, which indicated that eutectic Si had been well refined (Figure 7d,e). After 10 h of the second solid solution process (Figure 7f), the passivation of the primary Si no longer had sharp edges. The eutectic Si is spheroidized and coarsened. The coarsening of eutectic Si affects the solution effect and adversely affects the mechanical properties of the alloy [28,29].

**Figure 8:**
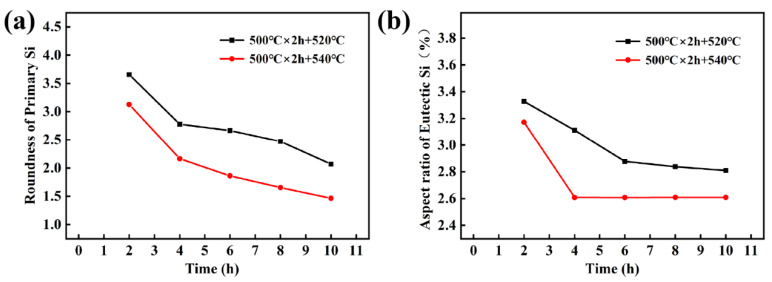
The statistics of Eutectic Si of Al-Si alloys with different SHT: (**a**) R_a_ of Primary Si, (**b**) Aspect ratio of Eutectic Si.

Figure 8a,b show detailed statistics on R_a_ of the primary Si and eutectic Si aspect ratios, respectively. The curves decrease faster in the second stage SHT at 540 °C. The atomic diffusion rate increases at high temperatures. Owing to the concentration gradient, elements can quickly dissolve into the matrix, which passivates and spheroids the Si phase. As shown in Figure 8a, the minimum R_a_ value was 1.46. As R_a_ tends to one (with progressively blunter edges), the morphology of the particles tends to become spherical. The risk of stress concentration at the tip and corner of the primary Si crystal on the mechanical properties of the alloy was reduced [30]. As shown in Figure 8b, the aspect ratio of eutectic silicon has a minimum value of 2.56. However, further extension of the second solid solution time induced coarsening of the eutectic Si phase at 540 °C, worsening the solid solution effect. The fragmentation and coarsening of eutectic Si occur simultaneously, which together determine the aspect ratio of eutectic Si. Increasing the solid solution temperature accelerates the coarsening of eutectic Si, leading to an increase in the aspect ratio of eutectic Si [31]. At lower solid solution temperatures, both spheroidization of eutectic Si and passivation of primary Si require a longer SHT time to achieve excellent solution results. In contrast, with an increase in the solution temperature, it only takes a short time to change the microstructure. The trend in the aspect ratio of eutectic silicon indicates that the Si particles experience spheroidization during the solid solution phase, which is a typical maturation process. The total number of particles is decreasing, and the interfacial energy is minimized by the gradual engulfment of smaller particles by larger ones. Due to the solid solution process, the Si element in the matrix can be dissolved into the matrix through the concentration gradient, making the Si phase gradually spheroidized. At the same time, the spheroidization of the Si phase can reduce the interfacial energy of the Si phase and the matrix. As the solid solution process proceeds, the solubility of elemental Si in the matrix reaches saturation, so the aspect ratio gradually tends to level off after 4 h.

**Figure 9:**
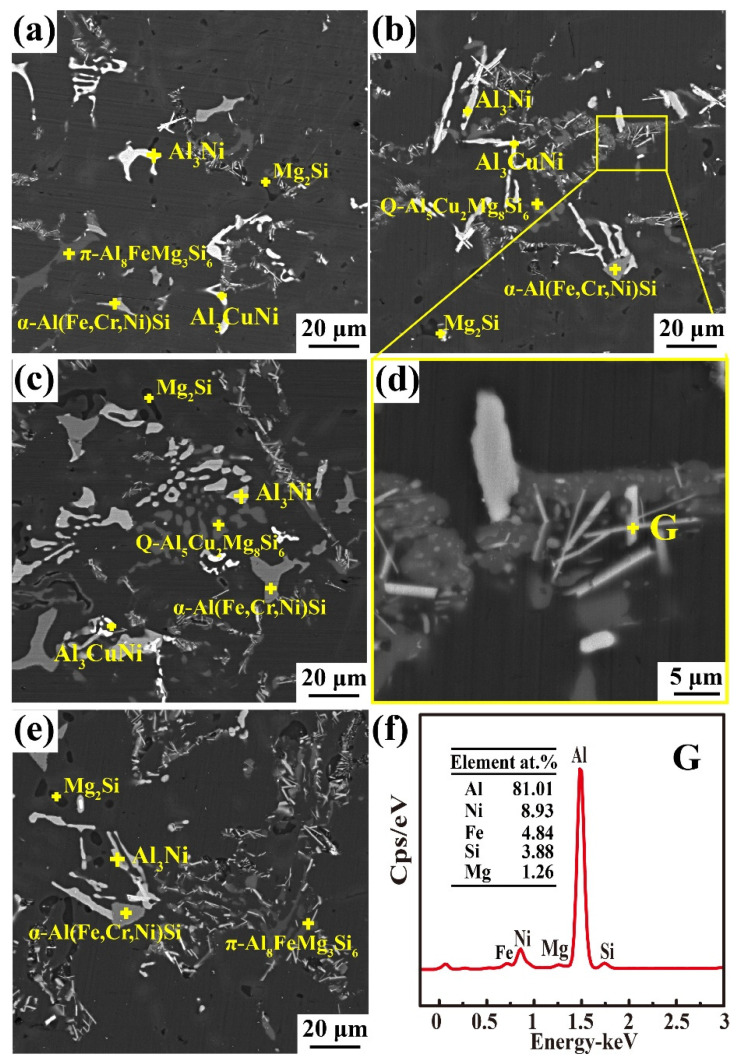
SEM images of Al-Si-Cu-Mg-Ni alloy under different solid solution conditions: (**a**) 500 °C for 2 h and 540 °C for 2 h, (**b**) 500 °C for 2 h and 540 °C for 4 h, (**c**) 500 °C for 2 h and 540 °C for 8 h, (**d**) enlargement of the boxed area in (**b**), (**e**) 500 °C for 2 h and 540 °C for 10 h, (**f**) EDS analysis of Point G in (**d**).

Figure 9 shows the backscattered electron images of the Al-Si-Cu-Mg-Ni-Cr alloy after the second stage SHT at 540 °C for various times. As the second solid solution time increased, the black Mg_2_Si phase showed a short rod and spherical shape, which was different from the as-cast skeleton shape. The off-white Al_3_Ni phase was blocked for 2 h (Figure 9a) in the second solid-solution step before gradually dissolving partially into short rods. Because the interfacial energy between the Q phase and the α-Al phase decreases, the Q phase dissolves from a block to a sphere (Figure 9b,d). The Al_3_CuNi phase fragmented into bright white short rods (Figure 9d). The bright grey herringbone α-Al (Fe, Cr, Ni) Si and dark grey π-Al_8_FeMg_3_Si_6_ phases did not exhibit any significant change in morphology (Figure 9e). As shown in Figure 9c, short white needle-like phases appeared in the Q and π phases. In Figure 9f, Al_9_FeNi is the emerging phase, as determined by EDS analysis. Ni can transform Al_5_FeSi into the Al_9_FeNi phase via an inclusion crystal reaction. When heated for SHT, the Al_9_FeNi phase was more easily broken and fragmented. Concurrently, as a thermodynamically stable phase below the solidus, Al_9_FeNi can remain intrinsically in the microstructure after the second solid-solution treatment. In addition, Al_9_FeNi plays an important role in improving thermal stability at 450 °C [16,32,33,34,35].

**Figure 10:**
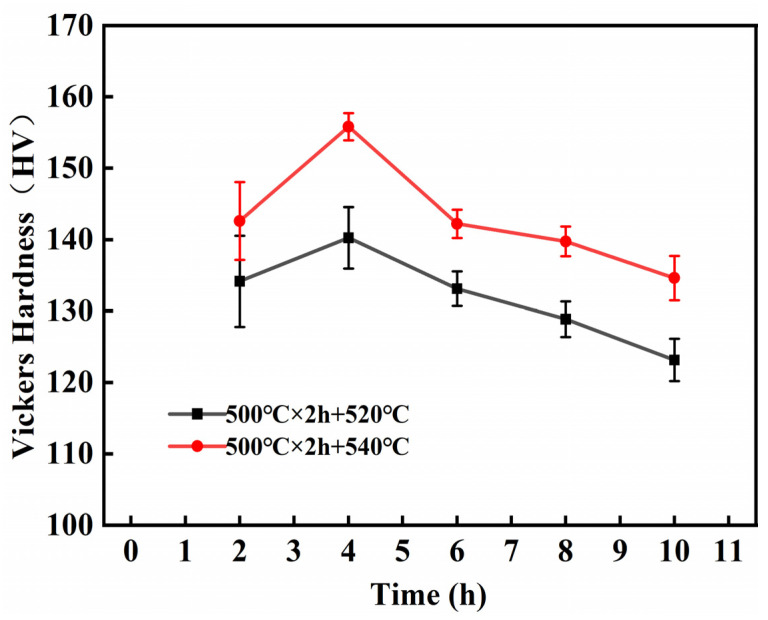
Microhardness of Al-Si alloy at different solution-treated conditions.

The microhardness of Al-Si-Cu-Mg-Ni-Cr alloy after two-step solution and ageing at 180 °C for 4 h was shown in Figure 10. The microhardness of the alloys tends to increase and then decrease at both SHT temperatures with the increase in second solid solution time. As the solid solution temperature increased, the microhardness of the alloy increased at 540 °C for the same solid solution time. This is because an increase in the solution temperature increases the solution degree, which leads to lattice distortion and increases dislocation resistance. Simultaneously, the grain size decreases with increasing phase change drive and nucleation rate [36,37]. With the continuous increase in solution time, the phase transformation of molten and spheroidized eutectic Si becomes coarse, which leads to grain growth and adversely affects microhardness. Therefore, the maximum microhardness of 155.82HV produced under the optimum solution conditions of 500 °C for 2 h and 540 °C for 4 h is much higher than that of as-cast alloy of 85.93 HV.

### 3.2. Optimization of the Ageing Process

**Figure 11:**
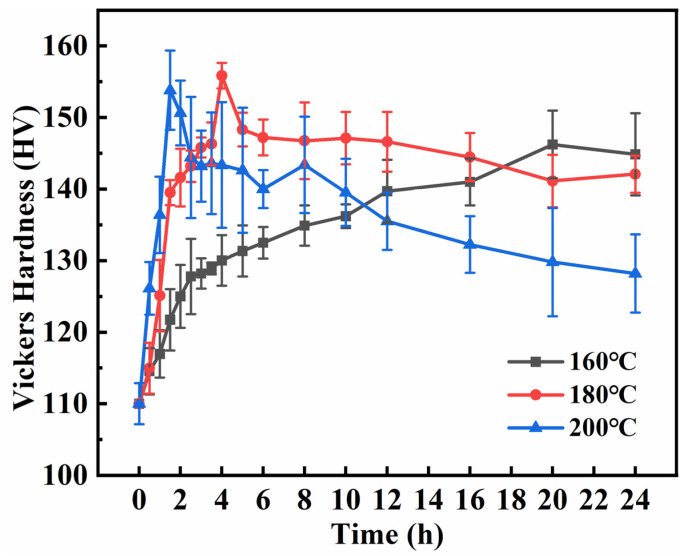
Effect of ageing time at different temperatures on microhardness of Al-Si alloy.

Figure 11 shows the relationship between microhardness and ageing time. The alloy exhibited significant strengthening during ageing. As the ageing temperature increased, the time required for the alloy to reach peak microhardness decreased. The hardening curve at 180 °C has only one microhardness peak value of 155.82 HV at 4 h. With an increase in ageing time, the microhardness curve decreases marginally and then tends to be stable, indicating that the phase transformation is more significant. The hardening curve took a longer duration of approximately 20 h to reach the peak value of 146.22 HV at 160 °C. At the ageing temperature of 200 °C, the alloy has two peaks; it reached the first peak value of 153.8 HV in approximately 1.5 h, and then the second peak value of 143.72 HV in 8 h. The microhardness of the alloy tends to decrease with the extension in time. During ageing, the movement of solid solution atoms is dominated by a diffusion mechanism, following the Arrhenius equation in Equation (2):

(2) $$D=D_{0}\exp[Q/(RT)]$$

where *D*_0_ is the diffusion constant, Q is the activation energy, R is the gas constant, and T is the thermodynamic temperature [11].

The rate of diffusion increased at higher temperatures. The de-solvation process in the supersaturated solid solution treatment required less time. In addition, solute atoms migrated to the precipitated phase in less time. After peak ageing, with the extension in time, the precipitation phase in the alloy coarsens and the supersaturation concentration decreases, resulting in deterioration of the solid solution strengthening effect of the alloy. Consequently, the microhardness value of the curve decreases. Therefore, the optimum ageing condition was 180 °C for 4 h.

**Figure 12:**
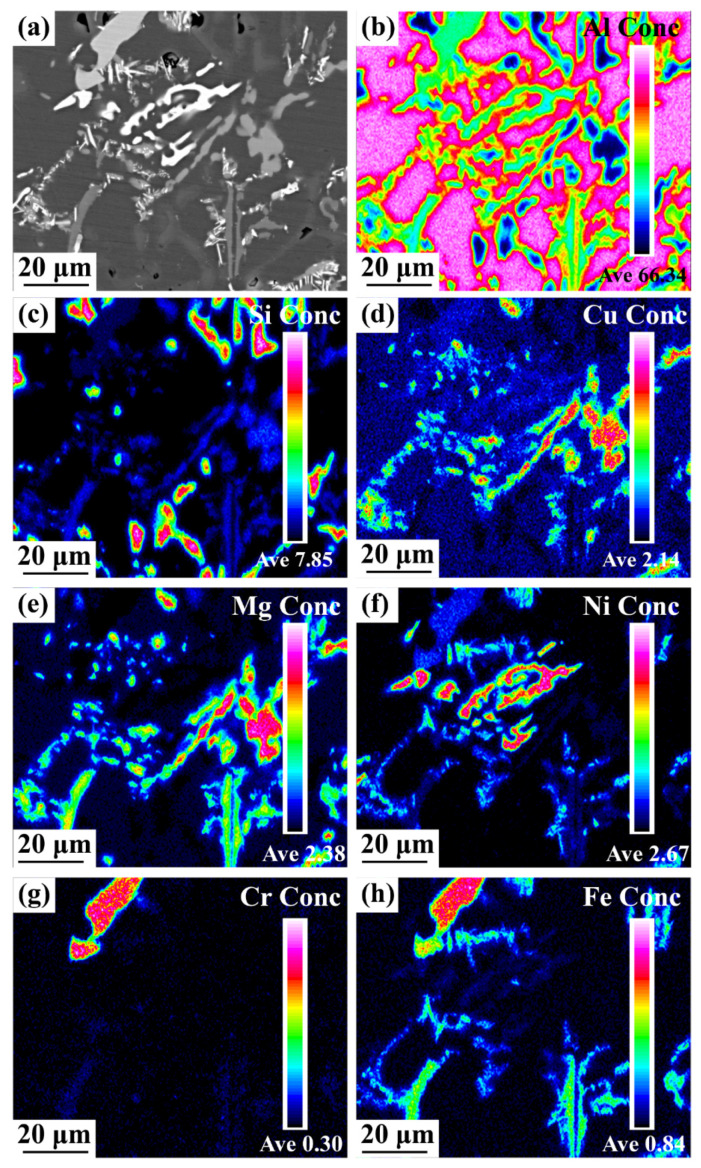
EPMA images and elemental mapping of the T6 state alloy: (**a**) alloy intercept, (**b**–**h**) elemental distribution.

Figure 12 presents the EPMA images of the alloy after 4 h of ageing treatment at 180 °C. The spatial distribution of the main alloying elements (Al, Si, Cu, Mg, Ni, Cr, and Fe) is included. In Figure 12d,e, the reformed Cu and a few Mg elements appear in the same position in the matrix as the Q phase. In addition, a few Mg elements formed Mg_2_Si with Si (Figure 12c,e). Ni was mainly used to form the Al_3_Ni phase (Figure 12f), and a few Ni and Fe elements formed the Al_9_FeNi phase (Figure 12f,h). In addition, Cr was still mainly involved in the formation of the α-Fe phase (Figure 12g,h). Further, Fe was observed in the π phase (Figure 12h). Comparing with the SEM image of the as-cast alloy in Figure 6, it can be found that the alloy re-precipitates into a fine, diffusely distributed second phase after heat treatment, which includes the Cu-containing phase, Mg_2_Si phase, and Ni-containing phase. This is due to the element segregation caused by the unbalanced solidification of the alloy during melting, which makes the unbalanced microstructure appear. Solution treatment makes the distribution of alloy elements uniform and reduces the unbalanced structure in the alloy. The eutectic Si gradually spheroidizes and some primary phases gradually dissolve. Cu, Mg, and Ni elements diffuse into Al matrix, forming complex supersaturated solid solution. After ageing treatment, the alloy elements dissolved into the matrix are reprecipitated into a fine and dispersed second phase in the form of dispersed phase.

### 3.3. Mechanical Properties

**Figure 13:**
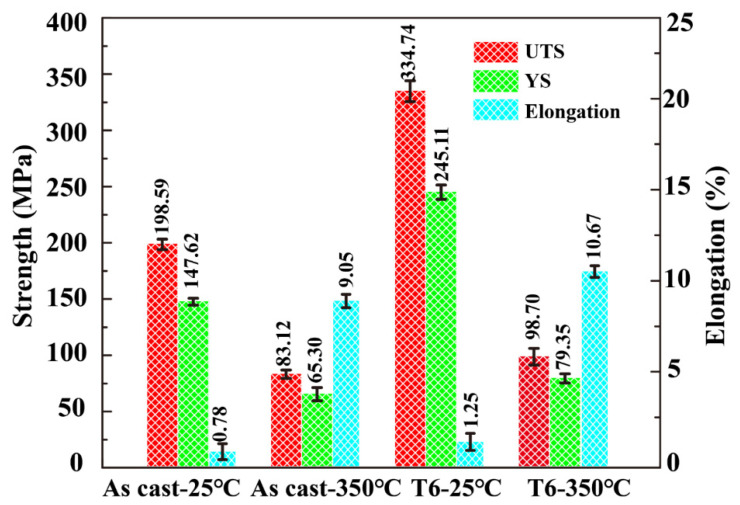
Tensile results for as-cast and T6 alloys at 25 °C and 350 °C.

Figure 13 provides a graphical representation of the tensile property data of the as-cast and T6 alloys at room temperature and 350 °C. At peak of ageing, the yield strength (YS) and e ultimate tensile strength (UTS) of the alloy were significantly improved. At room temperature, the UTS of the T6 state was 334.75 MPa, yield strength was 245.11 MPa and elongation was 1.25%. Compared to the as-cast alloy, the mechanical properties such as UTS, yield strength, and elongation of the T6 alloy at room temperature increased by 68.56%, 66.04%, and 37.60%, respectively. At 350 °C, the tensile strength, yield strength, and elongation of the T6 alloy were 98.70 MPa, 79.35 MPa, and 10.67%, respectively, which were 18.74%, 21.51%, and 15.18% higher than those of the as-cast alloy, respectively. After T6 treatment, the mechanical properties of the alloy were significantly improved owing to the morphological amelioration of the Si phase (Figure 7) and the spheroidization of the Al_3_CuNi and Al_3_Ni phases (Figure 9). With an increase in temperature, the control of the thermal activation of the lateral slip decreases the ultimate strength and yield strength, which tend to move dislocations. However, the thermal stability of the Q phase decreases at 350 °C. The Al_3_Ni, Al_3_CuNi, and Al_9_FeNi phases can maintain high thermal stability, facilitating the T6 state alloy in excellent high-temperature performance [32]. 

As shown in Table 2, in comparison with the pioneer studies, it was found that in this paper, with the addition of 0.2 wt.% Cr and the optimized heat treatment process, the UTS could reach 98.7 MPa at a reinforcing element of 1 wt.% Cu and Ni. Table 3 shows the material variations and types of tests in this paper.

**Figure 14:**
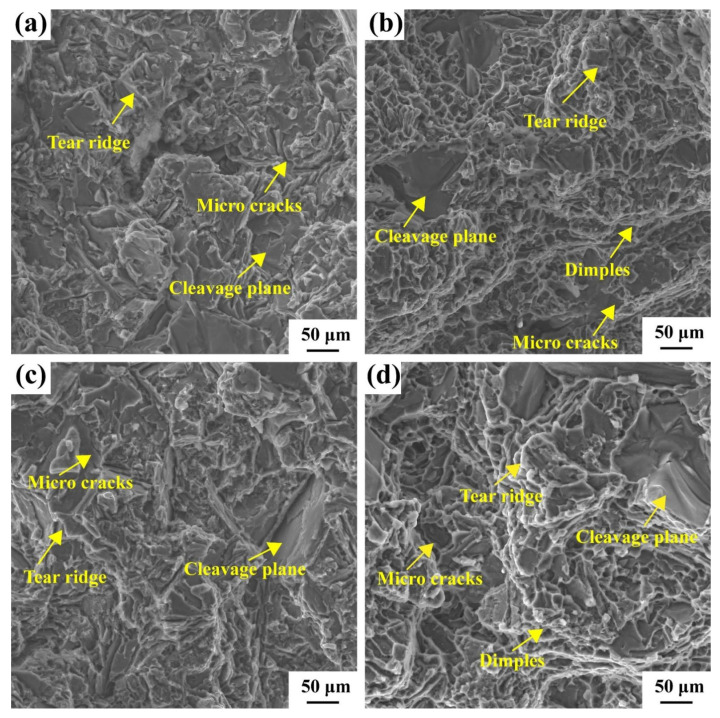
SEM micrographs of the typical fracture surface of the (**a**) as-cast alloy at 25 °C, (**b**) as-cast alloy at 350 °C, (**c**) T6 state alloy at 25 °C, (**d**) T6 state alloy at 350 °C.

Figure 14 shows the fracture surfaces of the alloys studied after tensile testing at room temperature and 350 °C. After tensile testing at room temperature, the fracture surfaces of the as-cast and T6 alloys were intergranular, as shown in Figure 14a,c. In addition, microcracks tend to initiate brittle intermetallic compounds. The strong interaction between the plastic or dislocation slip band and dispersed phase precipitation, particularly at the grain boundary, leads to intergranular fracture. Figure 14b,d show high-temperature tensile fractures at 350 °C. Quasi-cleavage can still be observed on the fracture surfaces of the cast and T6 alloys. This indicates that the fracture mode of the alloys was a mixture of brittle and ductile fractures at 350 °C. The alloy exhibited numerous small dimples and tearing edges on the alloy port. A typical ductile fracture is the formation, growing, and coalescence of micro-voids caused by cracking during diffusion of the second phase. The interface between the precipitating phase and ductile matrix broke when the interfacial stress exceeded the critical stress, resulting in the formation of cavities. In addition, defects such as voids generated during the casting process are the preferred locations for crack initiation.

**Figure 15:**
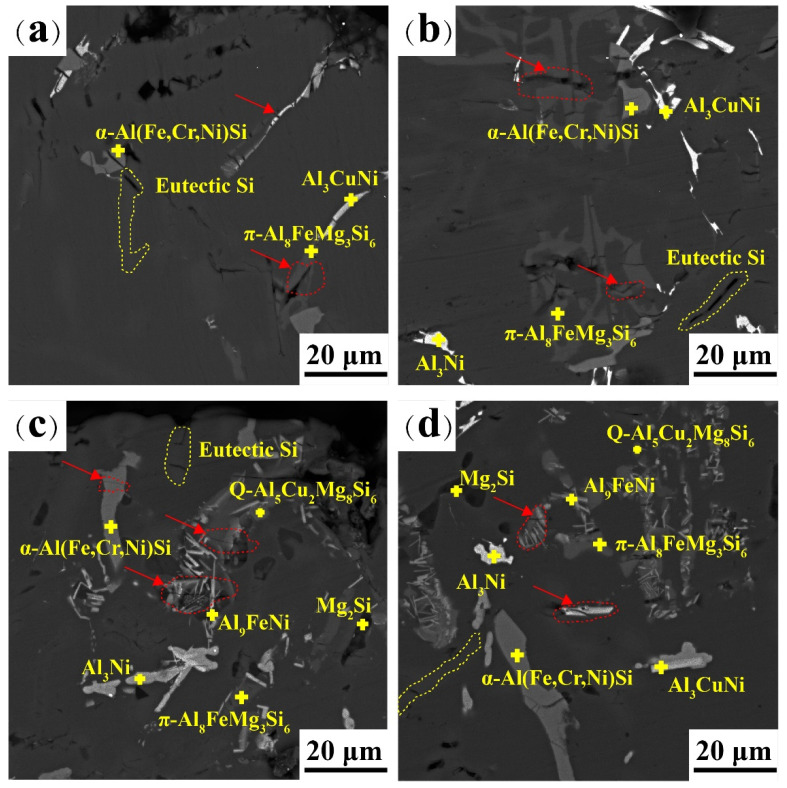
SEM images demonstrating the overall view of the polished cross-section of the tensile tested samples near the fracture surface of (**a**) as-cast alloy at 25 °C, (**b**) as-cast alloy at 350 °C, (**c**) T6 state alloy at 25 °C, (**d**) T6 state alloy at 350 °C.

SEM micrographs of the tensile fracture surfaces of the as-cast and T6 alloys at different temperatures are shown in Figure 15. The load transfer depends on the Al matrix, eutectic Si, and intermetallic compounds. Microcracks appear at different test temperatures (indicated by red arrows). In Figure 15a,c, microcracks span the intermetallic compounds and eutectic Si phases. Furthermore, the particles cracked several times without debonding. This shows that a strong interfacial bond exists between the particles and the matrix at room temperature. As shown in Figure 15b,d, the solid softens and loses its general properties at higher temperatures. The crack extended along the interface between the intermetallic compound and the matrix. The accumulation of dislocations at the interface between the particles and the matrix leads to debonding [38,39]. In Figure 15c, the cracking of the T6 alloy is concentrated in the eutectic Si and α-Fe phases after drawing at room temperature. The Ni-rich, Q, and Mg_2_Si phases, as strengthening phases, have rare cracks. In Figure 15d, few cracks are observed in the Al_3_Ni and Al_9_FeNi phases, indicating that they can inhibit cracks at 350 °C. Furthermore, the width of the crack opening increased significantly with increasing temperature.

## 4. Conclusions

The evolution of the organisation and mechanical properties of Al-Si-Cu-Mg-Ni-Cr alloys subjected to different heat treatments was investigated. The main conclusions are as follows.

(1) In the as-cast state, the alloy consists of an α-Al matrix, massive primary Si, needle-like eutectic Si, dark grey Chinese-script π-Al_8_FeMg_3_Si, light grey fishbone α-Al (Fe, Cr, Ni) Si, black skeletal Mg_2_Si, grey–white reticulated phase Q-Al_5_Cu_2_Mg_8_Si_6_, white needle-like phase δ-Al_3_CuNi, and white skeletal phase ε-Al_3_Ni phase.
(2) With an increase in the temperature and time of the second step of SHT, a new white short rod-like Al_9_FeNi phase is formed in the alloy. Primary Si passivation is evident, and eutectic Si also fuses and spheroids. The second phase of the reticular skeleton gradually fused into short rods; however, the morphology of the π-Fe phase and α-Fe showed no significant change. According to R_a_ of the primary Si, aspect ratio of the eutectic Si, and microhardness test, the optimum SHT conditions were 500 °C for 2 h and 540 °C for 4 h.
(3) Under the optimised solid-solution process at 500 °C for 2 h and 540 °C for 4 h, the ageing temperature and time were further optimised. The microhardness of the alloy initially increased. After reaching the maximum microhardness, the microhardness decreased with an increase in ageing time. The optimum ageing process was 180 °C for 4 h and the microhardness of the alloy reached a maximum value of 155.82 HV.
(4) The tensile test results at different temperatures show that the mechanical properties of the alloy were significantly improved after T6 heat treatment. When the heat-treated alloy is stretched at room temperature, the Ni-rich, Q, and Mg_2_Si phases play a major role in strengthening. The Al_3_Ni and Al_9_FeNi phases inhibited cracking at 350 °C. As the temperature increased, the fracture mechanism changed from quasi-cleavage to the coexistence of quasi-cleavage and dimples. At the end, optimizing the high temperature properties of piston materials is an important area of scientific and technical research. Future work could attempt to add rare earth elements to the Al-Si-Cu-Mg-Ni-Cr alloy to further improve the elevated temperature mechanical properties of the alloy.

## Figures and Tables

**Table 1:** Chemical composition of the alloy (wt.%).

	Si	Cu	Mg	Ni	Fe	Ti	Cr	Al
Alloy	12.5	1.14	1.29	1.15	0.39	0.13	0.18	Bal.

**Table 2:** Tensile properties of T6 alloys at 350 °C.

Alloy	Heat Treatment	UTS at 350 °C/MPa
Al-12.5Si-5Cu-2.0Ni-0.84Mg-0.24Cr [21]	480 °C × 3 h + 200 °C × 8 h	77.20
Al-13.0Si-3.7Cu-3.2Ni-1.1Mg-0.5Cr [23]	490 °C × 3 h + 200 °C × 8 h	98.61
Al-13.1Si-1.08Cu-1.0Ni-1.05Mg [33]	490 °C × 3 h + 200 °C × 8 h	61.63
Al-12.5Si-1.14Cu-1.29Mg-1.15Ni-0.18Cr	500 °C × 2 h + 540 °C × 4 h + 180 °C × 4 h	98.70

**Table 3:** The alloy variations and testing types.

Alloy Variations	R_a_ of	Aspect Ratio	Micro-Hardness	Tensile Properties at 25 °C			Tensile Properties at 350 °C		
	Primary Si	of Eutectic Si		UTS/ MPa	YS/ MPa	Elongation/%	UTS/ MPa	YS/ MPa	Elongation/%
As cast	4.3	3.73	85.93	198.59	147.62	0.78	83.12	65.30	9.05
T6	1.46	2.56	155.82	334.74	245.11	1.25	98.70	79.35	10.67

## Data Availability

Data will be made available upon request.
